# Weaving Equity into the Fabric of Medical Research

**DOI:** 10.1007/s11606-022-07450-3

**Published:** 2022-03-01

**Authors:** Krisda H. Chaiyachati, Rinad S. Beidas, Meghan B. Lane-Fall, Katharine A. Rendle, Rachel C. Shelton, Elinore J. Kaufman

**Affiliations:** 1 Perelman School of Medicine, University of Pennsylvania, Philadelphia, PA USA; 2 Department of Sociomedical Sciences, Columbia University Mailman School of Public Health, New York, NY USA

Health equity is the principled commitment to reduce and eliminate health inequities and their determinants.^1^ Contemporary social movements and the longstanding injustices spotlighted by the COVID-19 pandemic have brought renewed global attention to health equity. The attention towards understanding and improving the roots of health disparities at a population level is not new—decades (if not centuries) of research has been conducted by scholars in other fields like public health.^2^ This attention, however, has largely been placed outside the historical focus of the medical research community. However, medical research, particularly research the kind that involves people (e.g., human subjects research), plays a pivotal role in generating evidence that promotes or impedes population-level health equity. For example, well-established racial disparities in asthma rates can be mitigated by delivering interventions via community health workers.^3^ In contrast, the historic incorporation (until recently) of race in equations used to estimate kidney function has contributed to differential rates of kidney transplants and access to specialists.^4^ The impact of COVID-19 and racial injustices of 2020 increased the medical community’s attention towards combating health inequities and their root structural and social determinants.

Increased attention and funding will undoubtedly shift some researchers to prioritize health equity in their work. However, the proportion of medical researchers that prioritize or integrate efforts to achieve equity within their work is likely to remain low, because many researchers may not routinely do so or know how to do so given the historic lack of available training.

Equity cannot be a niche pursuit for a select group of researchers. The history of medical research is rife with examples that knowingly or unknowingly created inequity; that reified social differences as biological ones; and that, too commonly, simply ignored health equity and its determinants.^5^ For example, unfounded assumptions about race-based biological differences led to the adoption and persistence of race adjustments in clinical tests such as glomerular filtration rate and pulmonary function testing, with recent research showing multiplicative downstream harms to Black patients.^6^ Persistent underrepresentation of Black and Hispanic patients in cancer clinical trials in contrast to the general population provides a powerful example of longstanding, unequal access to advanced care.

To achieve equity in medical research, we must prioritize equity in discovery and all along the clinical and translational pipeline. Any research on health—especially research involving human participants—must weave health equity principles into how it is designed, funded, carried out, disseminated, and implemented. Research at every stage must seek to answer questions that are relevant to and address their impacts on equity, and even preparations for clinical trials should identify the ability to appropriately represent patient populations equally. Without structural changes which fully integrate health equity considerations into all aspects of the research paradigm, today’s commitments to equity will be temporary and superficial at best, and research will continue to contribute to and reinforce inequities in health and health care.

We propose three necessary structural changes to achieve greater integration of health equity. While these changes may be most effective and synergistic if implementation is coordinated between funders, universities, and research teams, each of these individual organizations could feasibly implement them within their own spheres of influence to ensure that medical research, particularly human subjects research, reaches its potential to promote equity. First, funders of medical research should make equity an explicit component of the peer review process by requiring health equity plans in all research proposals and holding researchers accountable for what they propose. Proposals should set enrollment targets to match the sociodemographic profile of a population or disease of interest, if not focus on historically underrepresented populations (e.g., race, income, education).^7^ If underrepresentation is anticipated, a justification should be required. During the study period, reporting of sociodemographics, like current reporting required by the NIH, would not simply be a data collection mechanism. Instead, identified underrepresentation would require action plans to achieve more equitable reach. Similar approaches, for example, have been adopted by Clinical and Translational Science Award modules, requesting plans to promote participation of underserved populations or engage stakeholders. Other funders should broadly follow suit. Like the increasingly common requirement for dissemination and implementation plans, equity plans should be tailored to the specific project, as different study types require different criteria for monitoring and assessing their impact on equity. And, like other key grant elements, these plans should be scored separately and considered in overall impact scores.

Correspondingly, study sections should include equity experts who review and critique health equity plans. In some cases, this expertise will reside with the subject matter experts already included in the panel. In other cases, specific equity reviewers must be recruited with the relevant experience or training, like the selective inclusion of reviewers with content and/or methodological expertise otherwise missing from review panels. Such reviewers should have experience in equity-focused research and should be familiar with the relevant literature. Given the prestige associated with appointment to grant review panels, those who assemble such panels should consider inviting equity experts to be regular or “standing” members of such groups.

Second, health equity should be further integrated into human subjects research approval and review structures within institutions, including the Institutional Review Board (IRB) and Data & Safety Monitoring Board (DSMB). These formal oversight groups exist to guide and enforce central principles of science such as respect of persons, beneficence, clinical efficacy, and safety. While not explicit, equity underlies and informs each of these principles. IRBs already identify protected groups of research participants at high risk of coercion such as children, prisoners, or pregnant women. In the same spirit, IRBs should require researchers to submit an equity monitoring plan for all studies. Likewise, as DSMBs independently evaluate data accumulated during ongoing clinical trials to assess the risks and benefits to participants, DSMBs should also identify disparities that develop as interventions are implemented and when outcomes are measured. Even when the risks or harms measured across groups are not significant, inequities during a trial may result in differential impact or unintended consequences to groups such as racial minorities or based on sexual orientation. Identified disparities should trigger the same thoughtful dialogue, course correction, and pauses or terminations of trials that would occur for any other safety or ethical concerns. In the end, disparities are harmful and should be treated as such.

Third, universities, research institutions, and funding organizations must cultivate, support, promote, and retain individual experts in equity to guide the transformation of medical research. Individuals with expertise, allies, and those with lived experience must take on leadership positions with appropriate support (financial and administrative) and decision-making authority to shape how equity is integrated into the monitoring and conduct of biomedical research. Expertise in other important arenas of medical research—quality, safety, statistical methodology, dissemination, and implementation—has become invaluable for generating high-level medical research, whether they are core to the aims of a study, or not. Developing and supporting equity experts should be no different. We expect that many institutions may find this expertise already within their own ranks, and they must support, empower, fund, promote, and retain future scholars with this expertise. At a broader level, we propose that all individuals engaged in the conduct of medical research, particularly human subjects research, be required to complete foundational training and continue to demonstrate an understanding of the harm created by health disparities and opportunities to generate more equity, and it should be tracked by research institutions, no different than routinely required IRB and responsible conduct of research trainings.

Adding obligations, reporting requirements, or administrative burden will likely be met with resistance.^8^ Yet, these are the very structural changes needed to make health equity explicit in research, and to hold researchers and institutions more accountable in creating and tracking changes towards a more equitable research enterprise. Realistically, these proposals will be necessary but insufficient to achieve equity alone. However, by design, these proposals will motivate and support researchers who have not traditionally considered or incorporated health equity measures and metrics in their research towards being more attentive to and thoughtful about equity.

Structural racism and discrimination pervade all stages of research, including who designs, executes, participates in, benefits from, and bears the burdens of medical research, and these decisions play a pivotal role in health equity. Rather than retroactively addressing inequities once they have been institutionalized, we have an opportunity to address equity prospectively and proactively in research from the beginning—when funding, designing, and conducting research.

As members of the medical community who conduct human subjects research, we must undo structures that have led to the perpetuation and exacerbation of inequity, and we must resolve not only to uncover these harms, but to proactively prevent them. The time is now to make health equity a part of our everyday fabric.
